# Fréquence de l’hyperlipasémie au cours des fractures osseuses chez les patients suivis à l’Hôpital Général Militaire de Référence Kokolo

**DOI:** 10.11604/pamj.2020.37.314.25123

**Published:** 2020-12-04

**Authors:** Mamy Mbelu, Donatien Kayembe, Luc Mokassa, Jean-Jacques Malemba

**Affiliations:** 1 Service de Biologie Clinique, Cliniques Universitaires de Kinshasa, Kinshasa, République démocratique du Congo,; 2 Service d´Orthopédie, Cliniques Universitaires de Kinshasa, Kinshasa, République démocratique du Congo,; 3 Service de Rhumatologie, Cliniques Universitaires de Kinshasa, Kinshasa, République démocratique du Congo

**Keywords:** Fractures osseuses, lipasémie, Kinshasa, Bone fractures, lipasemia, Kinshasa

## Abstract

**Introduction:**

décrire le profil des lipases sériques chez les patients présentant une fracture osseuse et en rechercher les facteurs associés.

**Méthodes:**

il s´agit d´une étude transversale réalisée de juillet à octobre 2013 à l´Hôpital Général Militaire de Référence Kokolo (HGMRK). Etaient inclus, les patients admis pour fracture osseuse et qui avaient consenti de participer à l´étude. Le groupe contrôle était constitué des donneurs bénévoles de sang. Les paramètres d´intérêt étaient les caractéristiques démographiques des patients, la lipasémie, le lipidogramme, le siège et le nombre de fractures.

**Résultats:**

quatre-vingt-trois patients avaient été inclus dans l´étude, tous du sexe masculin. Leur âge moyen était de 35,8 ± 12,8 ans. Un peu plus de soixante-dix-huit pourcent (78,3%) des fractures étaient dus à un traumatisme par balle. Le fémur était le siège le plus fréquent des fractures (30%), suivi par les os de l´avant-bras (20%) et le cubitus (15%). La concentration sérique moyenne de la lipasémie était de 43,6 ± 2,9 UI/L (valeur normale: ≤38UI/L) chez les fracturés contre 30,3 ± 2,3 UI/L chez les sujets contrôles (p<0,0001).

**Conclusion:**

une hyperlipasémie a été constatée chez des victimes de fractures osseuses, à une fréquence significativement plus élevée que chez les sujets contrôles. Cette hyperlipasémie n´était pas associée à la présence du syndrome clinico-biologique d´embolie graisseuse.

## Abstract (en)

**Introduction:**

the purpose of this study was to describe the profile of serum lipases in patients with bone fractures and to investigate the associated factors.

**Methods:**

we conducted a cross-sectional study at the Kokolo Military General Hospital in Camp Kokolo (KMGHICK) from July to October 2013. All patients admitted with bone fractures who had agreed to take part in the study were included. The control group included voluntary blood donors. The parameters of interest were demographic characteristics, serum lipase, lipidogram, the site and the number of fractures.

**Results:**

eighty-three patients were included in the study, they were all male subjects. Mean age of patients was 35.8±12.8 years; 78.3% of fractures were due to gunshot-related trauma. Femur was the most common seat of fractures (30%), followed by forearm (20%) and ulna (15%). Mean serum lipase concentration was 43.6 ± 2.9 IU/L (normal value: ≤38IU/L) in patients with fractures compared with 30.3 ± 2.3 IU/L in the control group (p<0.0001).

**Conclusion:**

a significantly higher rate of hyperlipasemia was found in patients with bone fractures compared with the control group. Hyperlipasemia was not associated with clinicobiological fatty embolism syndrome.

## Introduction

La maladie thromboembolique veineuse (MTEV) est un problème de santé publique et l´une des principales causes de décès cardiovasculaire dans le monde [1-4]. C´est l´une des complications les plus fréquentes chez les patients hospitalisés. Les données épidémiologiques sur la MTEV sont rares, en Afrique subsaharienne [5,6]. Les estimations d´incidence et de mortalité de la MTEV sous ses deux aspects -à savoir la thrombose veineuse profonde et l´embolie pulmonaire- sont très variables.

L´incidence annuelle de la thrombose veineuse profonde serait de 120 pour 100,000 personnes en France et de 60 à 100 pour 100000 personnes à travers le monde [3]. L´incidence annuelle de l´embolie pulmonaire se situerait entre 60 et 111 pour 100,000 en France et entre 23 et 107 pour 100,000 dans le monde [1]. La MTEV serait responsable d´une mortalité déclarée en France de 7,2 pour 100,000 habitants mais, selon les estimations issues des séries autopsiques internationales, entre 0,8 et 1% des patients hospitalisés seraient concernés par une EP [1]. Divers facteurs sont reconnus comme susceptibles de favoriser la survenue d´une MTEV. Il s´agit notamment de certaines maladies cardiovasculaires, de l´immobilisation prolongée, de la grossesse, de la chirurgie du petit bassin et des cancers [7-9].

L´embolie graisseuse, quant à elle, est causée dans 90% des cas par des fractures osseuses. C´est une maladie dont le diagnostic est loin d´être aisé. C´est ainsi qu´un bon nombre de cas sont identifiés à l´autopsie. Dans notre milieu, diagnostiquer formellement une embolie graisseuse est encore plus difficile. C´est alors que l´élévation rapportée de l´activité lipasique au cours de l´embolie graisseuse nous conduit à la question de savoir si une hyperlipasémie pourrait, chez un patient victime d´une fracture osseuse, constituer un marqueur indirect de l´embolie graisseuse, même infraclinique. Une majoration de la production de la lipase pulmonaire a, en effet, été décrite au cours de l´embolie graisseuse [10,11]. Elle est un mécanisme naturel tentant de lyser l´embole en vue de maintenir la perméabilité de la circulation artérielle pulmonaire.

C´est ainsi que la présente étude avait pour objectifs de déterminer le profil des lipases sériques au cours des fractures osseuses chez les patients suivis à l´Hôpital Général Militaire de Référence de Kinshasa (HMRK) et d´en identifier les déterminants.

## Méthodes

Il s´agit d´une étude transversale qui a été menée dans le service de chirurgie de l´HGMRK, situé au camp Kokolo, dans la période de juillet 2013 à septembre 2014. Etaient éligibles, tous les patients hospitalisés au service de chirurgie de l´HGMRK pour fracture osseuse. L´échantillonnage était de convenance. Les patients ont été inclus de façon consécutive. Des donneurs bénévoles de sang, appariés suivant l´âge et le sexe, ont servi de groupe contrôle.

Les données ont été recueillies sur une fiche d´enquête préétablie. Les paramètres d´intérêt étaient: les caractéristiques sociodémographiques des patients (âge et sexe), la date de survenue de la fracture, le siège de la fracture, le nombre de fractures, l´état de stabilité du foyer fracturaire, l´importance du déplacement, le caractère fermé ou ouvert de la fracture, la présence d´un choc hypovolémique et des lésions viscérales, la présence éventuelle des manifestations cardio-pulmonaires.

Le laboratoire du service de biologie clinique des CUK a servi pour la réalisation des analyses biochimiques autres que la lipasémie. Cette dernière ainsi que l´amylasémie ont été réalisées au laboratoire de la faculté de sciences pharmaceutiques de l´Université de Kinshasa. Un prélèvement de 5ml de sang veineux a été réalisé, dont 3ml dans un tube sec pour la biochimie et 2ml dans un tube avec EDTA, chez tous les patients retenus dans l´étude. Le prélèvement a été effectué entre 7h et 10h du matin, chez des patients en position assise et à jeun depuis 12 heures. Les échantillons ont préalablement été centrifugés à l´aide d´une centrifugeuse de marque KOKUSAN H-36 à la vitesse de 3000 tours par minute pendant cinq minutes. Après décantage, les sérums ont été aliquotés dans des cryotubes.

Ceux-ci ont été disposés dans des cryobox, puis conservés dans un congélateur de marque SANYO, à la température de -80°C. Ces sérums ont servi à la détermination quantitative de la lipasémie, de l´amylasémie, des triglycérides, du cholestérol et des lipides. La détermination de l´activité lipasique a été réalisée par la méthode enzymatique colorimétrique au moyen d´un spectrophotomètre semi-automatique de marque Humalyser Primus 2000, avec le réactif du laboratoire Cypress diagnostics, code HBE09. Les valeurs normales sont ≤ 38 UI/l. La lipasémie des sujets contrôles a servi à valider la valeur du kit.

Le dosage de l´amylase a été réalisé par la méthode colorimétrique enzymatique, au moyen d´un spectrophotomètre semi-automatique de marque Humalyzer Primus 2000, à l´aide du réactif de la firme Cypress diagnostics. Les valeurs normales sont <90UI/l. Le dosage du cholestérol a été réalisé par la méthode enzymatique colorimétrique avec le réactif du laboratoire human. Les données de la présente étude ont été saisies grâce aux logiciels Microsoft Word et Excel 2010. Après vérification de leur cohérence elles ont été transposées sur SPSS pour les analyses statistiques. Les données quantitatives ont été présentées sous forme de moyenne ±écart-type pour les variables à distribution normale; et sous forme de médiane pour les variables à distribution asymétrique. Les variables qualitatives ont été exprimées sous forme de fréquences (%).

Le test t de student a été utilisé pour comparer les moyennes et le test de Chi-carré de Pearson pour comparer les proportions. La corrélation entre la concentration sérique des lipides et la lipasémie a été recherchée à l´aide du coefficient R de Pearson. Le test de régression logistique a été utilisé pour rechercher les déterminants de l´augmentation de la lipasémie. Le seuil de signification statistique était de 0,05. Les principaux résultats ont été présentés sous forme de tableaux et de figures. La présente étude a été approuvée par le comité éthique de l´Université de Kinshasa. Tous les patients inclus avaient donné leur consentement éclairé.

## Résultats

Quatre-vingts trois traumatisés ont été inclus dans l´étude. L´âge moyen de patients traumatisés était de 35,8±12,8 ans. Tous les patients étaient des hommes. Quatre-vingt-un patients (97,6%) étaient des militaires. Le Tableau 1 montre que 74% des patients avaient entre 20 et 50 ans.

**Tableau 1:** répartition des patients par tranches d'âge

Tranches d'âge (ans)	n(%)
≤20	1 (1,6)
21-30	30 (36)
31-40	25 (30)
41-50	15 (18)
51-60	7 (8,4)
61-70	3 (3,6)
>70	2 (2,4)

Les fractures osseuses avaient diverses localisations. La fracture du fémur était la lésion la plus fréquente (36%), suivie de celle de l´avant-bras (20%), comme on le voit dans le Tableau 2. La principale cause de fracture osseuse était le traumatisme par arme à feu (Tableau 3). Au moment de l´inclusion, près de 90% des patients étaient immobilisés depuis plus de 6 semaines (Tableau 4).

**Tableau 2:** siège des fractures chez les patients inclus dans l'étude

Sièges	N(%)
Fémur	30 (36)
Cubitus et/ou radius	17 (20)
Humérus	12 (15)
Tibia	7 (8)
Autres	17 (21)

**Tableau 3:** causes des fractures osseuses

Cause	N(%)
Balle	65 (78,3)
ATR	16 (19,3)
Bombe	2 (2,4)

**Tableau 4:** répartition des patients fracturés selon la durée d'immobilisation

Durée d'immobilisation (semaines)	N (%)
1-2	5 (5,7)
2-6	4 (4,5)
>6	74 (89,8)

Sur le plan biologique, on note que 43 patients (55%) avaient une lipasémie élevée (≥38UI/L). En revanche, l´amylasémie était normale chez tous. Le Tableau 5 montre que, par rapport aux sujets contrôles, le cholestérol sérique des traumatisés était significativement plus élevé (p=0,010). Par contre, concernant les triglycérides, aucune différence n´a été noté (p=0,266).

**Tableau 5:** variations du lipidogramme et des lipases sériques chez les traumatisés

Lipides	Patients	Contrôles	p
Cholestérol	177,9±54,8	146,9±59,3	0,010
Triglycérides	108,1±10,3	82,8±32,4	0,266
Lipase	43,6±2,9	30,3±2,3	<0,0001

* Les résultats sont exprimés en mg/dl

Il existait une corrélation positive significative entre, d´une part, les triglycérides (p=0,0076) et le cholestérol (0,045) sériques et, d´autre part, la lipasémie comme le montre le Tableau 6. Le niveau de corrélation était faible avec les triglycérides et plus fort avec le cholestérol sérique.

**Tableau 6:** corrélation entres les lipases sériques, la cholestérolémie et la triglycéridémie

Lipides		Lipasémie	
	Coefficient d'estimation	R2 (%)	p
Triglycérides	0,290	32,0	0,0076
Cholestérol	0,247	53,0	0,045

* Analyse de corrélation de Pearson

Le Tableau 7 rapporte le profil de la lipasémie en fonction du siège de la fracture. Il en ressort que pour ce qui concerne la fracture du fémur, la majorité des patients présentait une lipasémie normale. Pour tous les autres sièges les cas d´hyperlipasémie étaient plus nombreux. Mais la différence n´était pas statistiquement significative (p=0,097).

**Tableau 7:** profil de la lipasémie en fonction du siège de la fracture

Siège de la fracture	Lipasémie (UI/L)		
	≤ 38	> 38	Tous
Fémur	20 (68%)	10 (31,8%)	30 (36%)
Humérus	3 (20%)	9 (40,9%)	12 (15%)
Tibia et/ou péroné	1 (14%)	6 (26%)	7 (8%)
Cubitus et/ou radius	3 (20%)	14 (32%)	17 (21%)
Autres	7 (41%)	10 (59%)	17 (21%)

## Discussion

L´objectif de la présente étude était d´étudier le profil de la lipasémie chez les patients à risque d´embolie graisseuse pour cause de fracture osseuse. Quatre-vingt-trois fracturés avaient été recrutés. Tous les patients étaient des hommes. Cette exclusivité masculine est due au fait que l´étude a été menée dans un hôpital militaire et que la plupart des patients étaient justement des militaires blessés par balle. Dans tous les cas les hommes sont plus susceptibles aux fractures osseuses que les femmes de par leur plus grande mobilité et l´exercice des métiers ou activités à risque [12,13].

Plus de la moitié des patients concernés étaient victimes d´une fracture du fémur. Mimoz *et al*. ont avancé des chiffres de 90% [14]. On notera qu´il n´y a pas eu dans notre série, des cas de fracture multiples ni de fractures avec lésions viscérales. Ce sont là des situations qui, avec les fractures instables et choc hypovolémique, favorisent la survenue d´une embolie graisseuse [15,16]. Aucun des 83 patients fracturés n´avaient présenté un syndrome clinique suggestif d´une embolie graisseuse, du moins pendant la période de notre étude.

Il reste que 55,4% des patients avaient présenté une hyperlipasémie. Une majoration de la production de la lipase pulmonaire a, en effet, été décrite au cours de l´embolie graisseuse [10,11,17]. La grande question reste de savoir si cette hyperlipasémie observée témoigne réellement de la présence chez nos patients d´une embolie graisseuse, fut-elle asymptomatique. Le démontrer reste difficile. Néanmoins une étude sur un large échantillon des patients et des sujets contrôles pourrait permettre de confirmer ou non la tendance à l´élévation des lipases sériques chez les personnes victimes des fractures osseuses.

Parmi les troubles lipidiques associés à l´embolie graisseuse seules l´hypocholestérolémie et l´hypertriglycéridémie ont été recherchées et elles n´ont pas été retrouvées chez les patients avec hyperlipasémie. Une étude sur un échantillon plus large est requise pour confirmer cette observation.

## Conclusion

Une hyperlipasémie a été constatée chez des victimes de fractures osseuses, à une fréquence significativement plus élevée que chez les sujets contrôles. Cette hyperlipasémie n´était pas associée à la présence du syndrome clinico-biologique d´embolie graisseuse.

### Etat des connaissances sur le sujet

- L´embolie graisseuse survient généralement à la suite d´une fracture osseuse;
- Une augmentation des lipases sériques peut survenir en cas d´embolie graisseuse.

### Contribution de notre étude à la connaissance

- La présente étude a montré que plus de la moitié des personnes victimes de fractures osseuses présentaient une hyperlipasémie et que la valeur moyenne de la concentration sérique des lipases était significativement plus élevée chez les patients que chez les sujets contrôles;
- Une corrélation positive a été observée entre les lipases sériques et le titre des triglycérides et du cholestérol.

## Remerciements

Les auteurs remercient M. Aliocha pour les analyses statistiques.
